# The 14th International Conference on Human Retrovirology: HTLV and related retroviruses (July 1–4, 2009; Salvador, Brazil)

**DOI:** 10.1186/1742-4690-6-77

**Published:** 2009-08-17

**Authors:** Luc Willems

**Affiliations:** 1 Cellular and Molecular Biology, Agro-Bio Tech (FUSAG), Gembloux, Belgium and Interdisciplinary Cluster for Applied Genoproteomics (GIGA), University of Liège (ULg), Belgium

## Abstract

The "14th International Conference on Human Retrovirology: HTLV and Related Retroviruses" was held in Salvador, Bahia, from July 1^st ^to July 4^th ^2009. The aim of this biennial meeting is to promote discussion and share new findings between researchers and clinicians for the benefit of patients infected by human T-lymphotropic virus (HTLV). HTLV infects approximately 15–20 million individuals worldwide and causes a broad spectrum of diseases including neurodegeneration and leukemia. The scientific program included a breadth of HTLV research topics: epidemiology, host immune response, basic mechanisms of protein function, virology, pathogenesis, clinical aspects and treatment. Exciting new findings were presented in these different fields, and the new advances have led to novel clinical trials. Here, highlights from this conference are summarized.

## Society affairs

**Figure 1:**
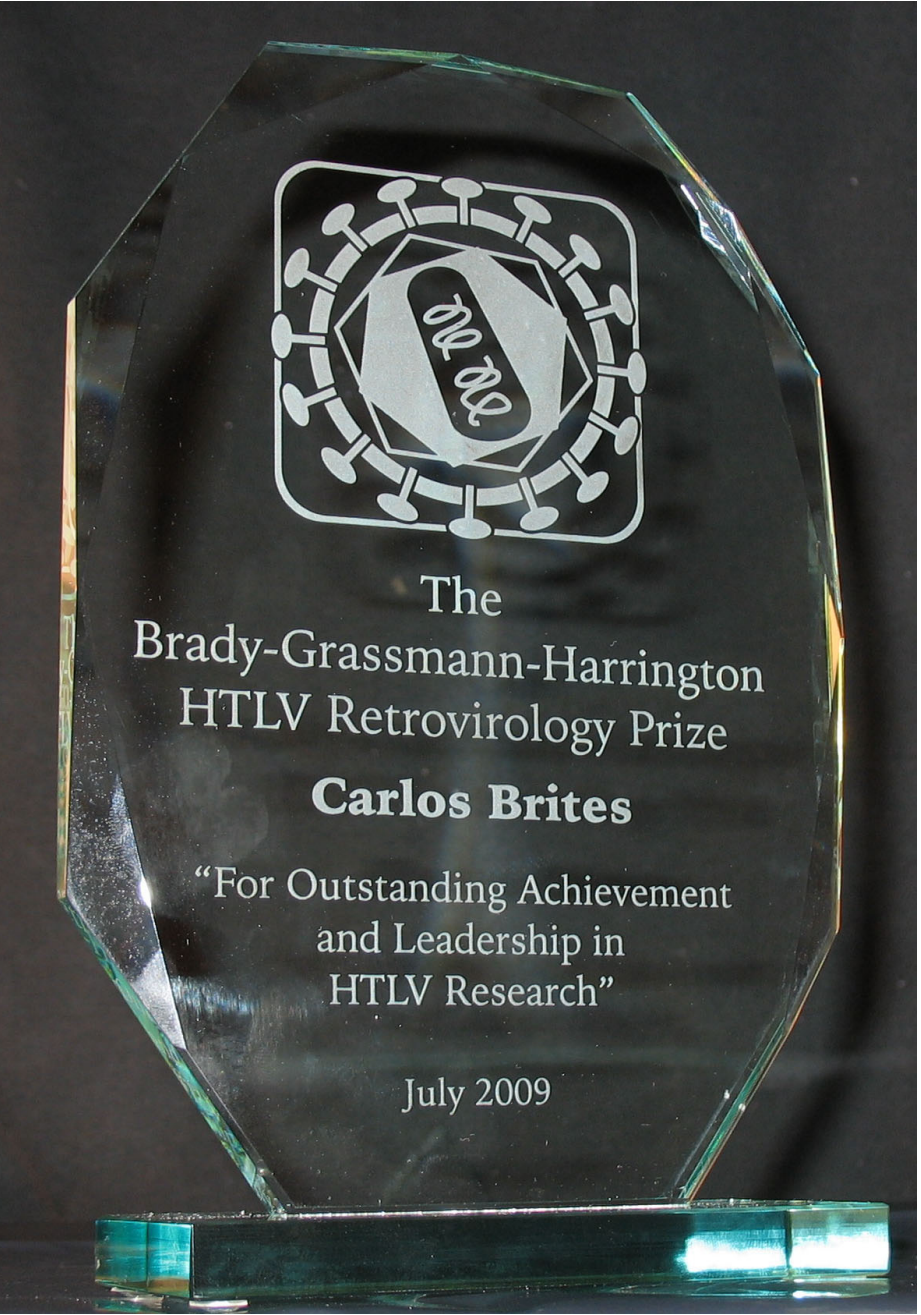
**The "Brady-Grassmann-Harrington prize" was awarded to Carlos Brites**.

In the opening ceremony, Carlos Brites (Salvador, Brazil), chair of the conference, underscored the importance of research in preventing HTLV-induced diseases in Brazil as well as throughout the world. Many physicians are not aware of the consequences of HTLV infection. HTLV-1 causes two major types of diseases: adult T-cell leukemia (ATL) and HTLV-associated myelopathy/tropical spastic paraparesis (HAM/TSP). Despite improved therapies, ATL still has a very poor prognosis and HAM/TSP has no satisfactory treatment. Graham Taylor, former president of the International Retrovirology Association, highlighted the key questions that each scientist or clinician should remember: "What do we know, what do we think to know and, ... what do our patients want us to know?". The meeting started with memorial lectures remembering three colleagues who departed us too early: John Brady, Ralph Grassmann and Bill Harrington. These three scientists were pillars of retrovirus research and made outstanding contributions to our understanding of HTLV-1 and patient care. The biennial HTLV Retrovirology prize was renamed the "Brady-Grassmann-Harrington prize" (Fig. 1) and was awarded to Carlos Brites (Salvador, Brazil) for his leadership and contributions to HTLV research. Later in the meeting, the association's McFarlane prize, which recognizes excellence in research, was awarded to William Hall (Dublin, Ireland) for his achievements.

## Findings

### Role of viral proteins in viral replication and pathogenesis

#### Accessory but important

In the keynote lecture, Genoveffa Franchini (Bethesda, USA), newly elected president of the International Retrovirology Association, focused on the role of accessory proteins in the development of HTLV pathogenesis. In addition to the classical structural, enzymatic and regulatory proteins, the HTLV-1 genome encodes a series of viral factors whose functions have been poorly understood [1]. In particular, Franchini reported that open reading frame 1 (ORF1) was required for infectivity in animal models. ORF1 encodes an uncleaved p12^I ^product that activates STAT5 signal transduction. A 8 kD cleaved form of p12^I^, p8^I ^is involved in T cell receptor (TCR) downregulation though inhibition of LAT (Linker for Activation of T Cells) accumulation at the virological synapse. LAT is known to interact with the TCR that binds the MHC during cell conjugation with the antigen presenting cell. Therefore, p8^I ^and p12^I ^have opposite effects on cell proliferation. Franchini showed that p8^I ^was transferred from the initial infected cells to the recipient T cells within minutes, and increased the adhesiveness of cells via LFA-1. The transfer occurred through nanotubes emerging from infected cells (MT-2) to uninfected Jurkat cells. p8^I ^increased tunneling nanotube formation. Non-infected cells were labile until they were touched by these nanotubes. Whether the virus is transferred through these structures is presently unknown. Cytotoxic T cells (CTL) were also recruited by the nanotubes and were anergized by p8^I^. This mechanism could lead to the inhibition of cytotoxic cell killing.

Another important accessory open reading frame (ORFII) encodes p30^II ^[2] and p13^II^. In the presence of Tax, p13^II ^is stabilized and localizes to the nucleus. Franchini reported that p13^II ^induced Tax degradation and inhibited it's transcriptional activity, thereby decreasing viral replication. In contrast, p13^II ^was stabilized in the presence of Tax through a mechanism that involved ubiquitination. Vincenzo Ciminale (Padua, Italy) described another role of p13^II ^that included the induction of mitochondria swelling due to insertion into the inner membrane [3]. Ciminale showed that p13^II ^induced a dose-dependent depolarization of the mitochondrial membrane and O_2 _consumption. Reactive oxygen species (ROS) production measured by Amplex red was increased by p13^II^. Expression of p13^II ^decreased the tumor growth in mice but activated primary PBMCs. This dual regulatory function illustrates the "ROS rheostat" theory that postulates that minimal levels of ROS are required to initiate cell proliferation, but that an excess ROS level induces apoptosis.

#### Antisense-strand encoded factors

A special lecture presented by Becca Asquith (London, England) was entitled "HBZ binding to HLA class 1 determines the outcome of HTLV-1". The goal of this project is to test the hypothesis that CD8 efficiency is determined by the binding of HLA class 1 molecules. Asquith synthesized peptides spanning each of the HTLV-1 proteins and tested them for binding affinity. In this manner, she validated the epitope predictions made by an in silico computer program (METASERVER). HLA-alleles that were HTLV-protective appeared selectively to bind HBZ more efficiently when compared to other viral proteins. In addition, HLA class 1 alleles from asymptomatic HTLV carriers (AC) had a better binding to HBZ when compared to alleles from HAM/TSP individuals. Asquith suggested that HLA-binding to HBZ is associated with a reduced proviral load. The most likely mechanism is that HBZ inhibits expression of other HTLV-1 genes allowing escape from the host immune response.

On the other hand, HBZ expression drives infected cell proliferation conferring a survival advantage to HBZ-expressing cells [4]. Masao Matsuoka (Kyoto, Japan) underlined this key role exerted by HBZ in development of ATL [5]. HBZ expression increased cell proliferation and induced T cell lymphoma in transgenic mice. Patrick Green (Columbus, USA) explained that HBZ interacted with CBP/p300 and formed heterodimers with CREB. HTLV-infected cells transfected with a HBZ-specific shRNA lentiviral vector proliferated slower and did not induce tumors in nude mice. In contrast to Tax, HBZ expression directly correlated with proviral loads. ATL cells from patients and tumor cells from transgenic mice were found to express FoxP3, a marker of T regulator cells (Tregs). In HBZ transgenic mice, increased proliferation of Tregs inhibited transcription of IL-2 through NFAT. This inhibition was antagonized by c-Fos indicating that HBZ replaces c-Jun in the complex. HBZ-Tg mice were observed to have impaired immune response to Listeria monocytogenes due to a lack of interferon (IFN) induction.

These recent data underscore an essential function of HBZ in HTLV-1 pathogenesis and an intricate interplay between HBZ and Tax [6]. HTLV-2 also encodes a protein from the complementary strand (Douceron et al, Blood in press). This protein was termed antisense protein HTLV-2 (APH-2) because it does not contain a bZIP domain and shares minimal homology with HBZ. Renaud Mahieux (Lyon, France) reported that APH-2 was not present in the nucleolus; it interacted with CREB but not p300, and it inhibited Tax transactivation. APH-2 and HBZ may thus be involved in transcriptional silencing of the virus in infected cells, a mechanism required for the virus to escape from the host's immune response.

#### The Tax oncoprotein

Besides antisense-strand encoded factors, the Tax oncoprotein plays an essential role in viral replication and pathogenesis [7]. Novel properties of Tax have been described at the conference. Susan Marriot (Houston, USA) reported that Tax deregulated checkpoints and interfered with repair of DNA strand breaks. In cells submitted to ionizing irradiation, Tax inhibited ATM phosphorylation and restricted the number of double strand breaks revealed by H2AX phosphorylation (γH2AX). Tax expressing cells thus fail to repair DNA damage after irradiation, possibly leading to genetic abnormalities.

Kuan-Teh Jeang (Bethesda, USA) outlined that Tax expression induced micronuclei resulting from trapping of chromosomes by nuclear envelope reformation after telophase. Jeang reported that chromosome missegregation can be seen in HTLV-cells probably due to a defect in their spindle assembly checkpoint (SAC). He discussed the two major checkpoints in mammalian cells: p53 at the G1/S junction and the SAC with its constituent mitotic arrest deficiency proteins (MAD) in the mitotic (M) phase of the cell cycle. He showed that mice knocked out (KO) for 1 allele of MAD1 (+/-) have a significantly higher probability of tumor development, and mice simultaneously with two checkpoint defects (MAD+p53) have highly increased chromosome instability. Jeang suggested that loss of two discrete checkpoints may lead to the emergence of multiple primary tumors in the same patient [8].

In the latter part of his talk, Jeang also addressed the current inability to transform human primary somatic cells using the HTLV-1 Tax oncogene. He showed that while differentiated human cells resist transformation by Tax, human stem cells could be efficiently transformed by Tax to give rise to tumor in immune-deficient mice. He then speculated that the seeds for human cancers may not be tissue somatic cells, but may instead be the tissue stem cells. This view converges with some (but not all) of the issues currently debated regarding the "cancer stem cell" hypothesis.

Tax activities are regulated by complex post-translational modifications, including phosphorylation (Françoise Bex; Brussels, Belgium) and ubiquitination (Journo et al, PLoS Pathogens, in press). In the cytoplasm, Tax activated NFκB by interaction with IKKγ/NEMO. Tax also bound to optineurin, a Golgi resident protein. Journo reported that optineurin stabilized Tax ubiquitination and enhanced Tax-dependent NFκB activation. Another Tax-binding protein, Tax1BP1, an ubiquitin adaptor for A20/Itch/RNF11 is involved in the same pathway.

Although increased proliferation of infected cells has clearly been observed in vitro as well as in patients, the mechanisms involved are still unknown. Tax is a major player in this process through interaction with the minichromosome maintenance protein (MCM2–7) complex (Mathieu Boxus; Gembloux, Belgium). Tax interacted and co-localized with MCM proteins in T lymphocytes. Moreover, Tax facilitated MCM3 binding to chromatin and increased the number of active replication origins during the synthesis phase of the cell cycle, thereby accelerating DNA replication. Silencing of MCM3 with shRNAs abrogated Tax-stimulation of replication origins. Tax also triggered re-replication, generating cells with > 4N DNA content. Replicative lesions activated the DNA damage response pathway, as revealed by phosphorylation of H2AX in cell lines established from ATL patients. These lesions can be converted into fatal replication lesions and aberrant mitosis (mitotic catastrophe) using DNA repair inhibitors, a strategy that may be useful for the treatment of ATL.

### Cell biology and host immune response

#### Infected cell types

CD4+ lymphocytes and to a lesser extent CD8+ T cells are considered as the main targets of HTLV-1. During his presentation, Francis Ruscetti (Frederick, USA) demonstrated that plasmacytoid dendritic cells (pDC) were highly infected by HTLV-1 in patients. In fact, all types of DC (pDC, myDC and MDDC) have been shown to be easily infected by HTLV-1 in vitro and efficiently transmit HTLV-1 to T cells [9]. Interestingly, Ruscetti found that the proviral load was higher in freshly isolated pDCs than in T cells. In both cell types, viral expression could not be detected at high levels in vivo. pDCs stimulated type I interferon α and β which interacted with their cognate receptors on virus infected cells and, through IFN-inducible genes, interfered with viral replication. In chronically infected pDCs, Ruscetti observed that IFN reduced the expression of HTLV-1. pDCs from ATL patients were found to be impaired in their response to TLR7 agonists and in their production of IFN-α. These observations supported a role for pDC in viral persistence and possibly ATL progression. Jean-Philippe Herbeuval (Paris, France) showed that HTLV-1 induces TLR-dependent immune response by pDCs. The pathway activated by HTLV-1 involved the acidification of the endosomes, the destruction of the virus, and the induction of the TLR. Inhibitors such as chloroquine and A151 (a TLR7 inhibitor) inhibited IFNα production and TRAIL expression on pDCs. Thus, there were two outcomes of infection of pDCs by HTLV-1: transmission to T cells or destruction in endosomes. Another regulatory TLR-independent mechanism of the innate immune response by Tax was described by Glen Barber (Miami, USA).

#### Mechanism of viral infection

Kathy Jones (Frederick, USA) in collaboration with Claudine Pique (Paris, France) reviewed the consecutive steps of virus infection involving heparan sulfate proteoglycans (HSPG), neuropilin 1 (NRP1, a receptor of VEGF165) and the glucose transporter (GLUT1) [10]. She pointed out that binding of HTLV-1 to NRP1 is first facilitated by HSPG. Consistently, enzymatic cleavage of HSPG was seen to decrease infection of DCs. In her model, NRP1 acted as a co-receptor of VEGF-R and increased HTLV SU binding to cells. A peptide spanning a KPXR consensus motif present both in SU (residues 90–94) and in VEGF blocked interaction with NRP1. Residue Arg 94 is known to be important for HTLV infectivity and belongs to a region targeted by neutralizing antibodies. The GLUT1 receptor is involved in a post-binding step. DCs also express a C-type lectin receptor called DC-SIGN which may be a target for antiviral therapy such as thieno-pyrimidines and tetrazolo-pyrimidines (Pooja Jain; Philadelphia, USA).

Using EM tomography, infection of T cells has previously been shown to occur through a virological synapse [11]. This process requires Tax expression, CREB activity and MEK-ERK signaling, and involves a polarization of the infected cell with the transmission of the virus to the target cell. Another interesting mechanism of infection was reported by Maria Thoulouse (Paris, France). In short term cultures of CD4+ cells from HAM/TSP, she saw that most viral particles were adhered to the outer part of the membrane and formed extracellular adhesive structures. These viral assemblies (called biofilms) were composed of particles embedded in the extracellular matrix that bridged an HTLV-1 infected cell and one or several target cells. She proposed that there are two independent routes for viral entry: transit through the virological synapse and endocytosis via biofilm structures.

#### Intracellular mediators

Andrea Kress (Erlangen, Germany) reported that increased levels of cAMP are present in long lived murine T cells and in ATL cell lines. In TESI cells derived from primary lymphocytes transduced with a Tax-expressing recombinant rhadinovirus vector, downregulation of Tax expression decreased the levels of cAMP. Elevated cAMP levels are due to downregulation of phosphodiesterase 3B (PDE3B) mRNA through epigenetic silencing. Whether higher levels of cAMP exert an immunosuppressive function remains an open question.

Ricardo Khouri (Salvador, Brazil) presented that HAM/TSP cells have decreased level of SOD1, which is involved in regulation of reactive oxygen species (ROS). He found that the SOD1 inhibitor D1 synergized with IFN-α but not AZT to induce apoptosis of infected cells. SOD1 may explain the efficacy of compounds such as vitamin C. ROS also appeared to be important mediators of BLV persistence (Amel Bouzar; Gembloux, Belgium). Indeed, spontaneous expression of ROS in short term cultures inversely correlated with proviral loads.

#### Antiviral cell response

The host immune response is believed to exert a tight control over the virus that continuously attempts to replicate [12]. Several cell types are involved in this process with CD8+ T cells being the best characterized. Yoshimi Akahata (Bethesda, USA) showed that degranulation (CD107a) and IFNγ expression is higher in HAM/TSP than in AC. CD244 is a lymphocyte activation molecule receptor that is highly expressed on CD8+ T cells. CD48, the ligand of CD244, inhibited spontaneous degranulation and IFNγ expression. Two associated proteins (SAP and EAT-2) were involved in signal transmission and CD8+ T cell response (IFNγ production). These observations demonstrated the involvement of the CD244-SAP signaling in HAM/TSP. As described for HTLV-1, Andre Oliveira (Dublin, Ireland) showed that HTLV-2 infected patients also have functionally competent CTLs.

The group of Mari Kannagi (Tokyo, Japan) provided evidence for the suppression of HTLV expression by stromal cells through a type I IFN response. The process was reversible since viral expression in infected cells was restored by their separation from stromal cells. The mechanism was reported to involve an interferon response since (i) an antibody directed against IFNβ blocked suppression by stromal cells and (ii) HTLV expression was suppressed in wild type but not in IRF-7 KO mice.

Invariant NKT (iNKT) cells were known to have anti-HTLV-1 activity (Yoshihisa Yamano; Kawasaki, Japan). These cells, which recognize and are activated by α-galactosylceramide (αGalcer) at the cell surface, connect innate and adaptive immune responses. The frequencies of iNKT were shown to be reduced in PBMCs from HAM/TSP and ATL patients compared to AC subjects. In PBMC cultures from AC but not ATL, stimulation of iNKT by α Galcer decreased the number of infected cells.

#### Chemokines and their receptors as potential novel therapeutic targets

CD4+CD25+CCR4+ cells are a major HTLV-1 reservoir. Interferon expression by Foxp3lowCD4+CD25+CCR4+ cells was found to be increased and correlated with HAM/TSP disease severity (Yoshihisa Yamano; Kawasaki, Japan). Removal of CD4+CD25+CCR4+ cells decreased the proliferation of CD4+ cells. Disease severity also correlated with the expression of CXCL10 and the soluble IL2 receptor. Fred Toulza (London, UK) indicated that the frequency of FoxP3+ CD4+ cells was increased in ATL. He found a negative correlation between the frequency of FoxP3+ CD4+ and the rate of CTL lysis. CCL22, the ligand of CCR4, correlated with Tax expression and FoxP3 frequency. CCL22 expressed by infected cells exerted chemoattraction of CD4+FoxP3+ and increased their viability.

Chemokine receptors may be potential targets for novel therapies. Indeed, inhibition of CXCR4 with AMD3100 suppressed the migration of ATL cells and murine lymphoblastoid cells from transgenic mice (Akira Kawaguichi, Sapporo, Japan). AMD3100 decreased phosphorylation of ERK by SDF1α and inhibits cell migration. Another example was CCR4 against which a humanized antibody (KW0761) has been designed (Dr. Utsunomiya; Kagoshima, Japan). The antibody exhibited high ADCC activity (antibody-dependent cellular toxicity) in cell cultures. In a phase I study, neutropenia and rash were the main side effects. Two partial and two complete responses were observed among 13 patients.

### Clinical manifestations and molecular epidemiology

In addition to HTLV-1, humans can be infected by 3 other members of the δ-retrovirus genus (HTLV-2, -3 and -4) [13-15]. HTLV-2 was first isolated from a patient with atypical hairy cell leukemia although further studies failed to confirm the association of HTLV-2 with lymphoproliferative diseases. This dogma was challenged by Ed Murphy (San Francisco, USA) who showed in a large (1,360 patients) and long term (18 years) survey that pneumonia, bronchitis and cancer were frequent in HTLV-2 infected patients. In fact, patients with HTLV-2 had more missed work days than patients with HTLV-1, indicating that HTLV-2 interferes with quality of life. Surprisingly, HTLV-2 infected subjects also had a significant shorter life expectancy. Murphy further described a constellation of diverse neurological manifestations associated with HTLV-1 and proposed the term "NASH" (neurological abnormality short of HAM). This study thus confirmed and extended pioneering observations reported by Abelardo Araujo (Rio de Janeiro, Brazil) who had challenged the restrictive concept stating that neurological signs are limited to HAM/TSP.

HTLV transmission has been a matter of intense debate. At first glance, it would appear that preventive measures prohibiting breast feeding are needed among HTLV-1 infected mothers. However, substitution with dry milk raises problems such as social habits, cost, availability of good quality water and protection against other pathogens. Recommendations by responsible pediatricians must individually take these parameters into account. As indicated by Soren Andersson (Stockholm, Sweden), it is important to keep in mind that, when studying HTLV, subjects may also be co-infected by other pathogens such as pulmonary tuberculosis. The group of Achilea Lisboa Bittencourt (Salvador, Brazil) provided evidence that infectious dermatitis (ID), a severe recurrent infected form of eczema in children, may represent a prodromal stage of ATL. Indeed, a proportion of ID subjects had monoclonal proviral integration and characteristic flower cells. Cases of HAM/TSP with ATL were unusually frequent in the region of Bahia. Uveitis in the intermediate uvea was also frequently observed in HTLV-1 infected patients. A poster from Daniel Ceccaldi's group (Paris, France) provided evidence using in situ hybridization that muscle cells were infected in 4 out of 12 patients with myositis. Patients had myositis-associated auto-antibodies and muscle specific CD8+ T cells.

Although HAM/TSP is usually a slow progressing disease, some patients exhibit a dramatic fast evolution. Eduardo Gotuzzo (Lima, Peru) described rapidly progressing HAM/TSP affecting 20% of Peruvian patients. Marco Lima (Rio de Janeiro, Brazil) previously evaluated a treatment with AZT and prenidoslone without any significant improvement in these patients.

Since the discovery of HTLV-1 three decades ago, apparently simple questions remain still unanswered: "Why do some subjects develop ATL and others HAM/TSP?"; "Why is there a predominance of females with HAM/TSP?" and "Why do some patients progress very rapidly?".

In contrast to HTLV-1 and -2, HTLV-3 and -4 have not yet been associated with any pathology; this is likely due to their recent identification and to the low number of available isolates. Three HTLV subtypes have closely related simian viruses (named STLV-1, -2 and -3) while a STLV-5 strain is presently still devoid of a human counterpart. Contrasting with the homogenous HTLV-1/STLV-1 genotypes, STLV-2 and HTLV-2 are quite distant and form two distinct groups. Therefore, it is impossible to discriminate between STLV-1 and HTLV-1 without knowing the origin of the sample. Antoine Gessain (Paris, France) presented recent data from Central Africa, where HTLV-2 is endemic in Bakola pygmies. Intriguingly, there was no HTLV-1 in pygmies, who were infected by HTLV-2 subtype B. This genotype was also found in Amerindians tribes from the region of Amazonia. These data support evidence for an ancient origin of HTLV-2 in Central Africa. Some unanswered questions remain: "Why is the seroprevalence in hunter-gathered Bakola Pygmies higher than Bantu farmers living in the same region?" and "How were pygmies infected by HTLV-2?". HTLV-3 is also found in Central Africa and is most likely transmitted from a variety of monkey species to humans during hunting or, alternatively, through intrafamilial transmission.

It thus appears that the PTLV family is composed of at least 5 genotypes. Although sequence divergence is more restricted, recent data show that this complexity also accounts for BLV where two new genotypes were described (Sabrina Rodriguez; Buenos Aires, Argentina).

### Therapy

#### Prospects for novel treatments of HAM/TSP

Animal models are important to understand the mechanisms of pathogenesis and to test novel therapies [16]. A strategy aimed at activating viral gene expression with valproic acid (VPA), a lysine deacetylase inhibitor, in order to expose virus-positive cells to the host immune response. The approach efficiently decreased the number of leukemic cells in BLV-infected sheep (Luc Willems; Gembloux, Belgium). The treatment has now been evaluated in a single-center, two-year open-label trial, with 19 HAM/TSP volunteers treated with oral doses of VPA (Stéphane Olindo; Fort-de-France, Martinique). The treatment did not alter the anti-viral CTL response and generated only minor side effects. Unfortunately, different parameters including the disability status scale, muscle testing score, Ashworth score, urinary dysfunction score and walking time test did not change significantly. Long term treatment with VPA was thus safe but did not alleviate the condition of HAM/TSP. Since the proviral loads before and at one year post-treatment were similar, long term VPA administration to early stage HAM/TSP patients should not be considered. A possible improvement of this strategy has been proposed by Renaud Mahieux (Lyon, France). He reported that a regimen combining VPA and AZT decreased proviral loads in STLV-1 infected baboons (Papio anubis). Whether this regimen is efficient in HAM/TSP remains to be tested.

Additional strategies have been proposed at the meeting including minocycline (an antibiotic that inhibits monocyte/macrophage activation; Yoshimi Akahata; Bethesda, USA), humanized mikβ1 (a monoclonal antibody against CD122, the β subunit shared by IL2 and IL15; Steven Jacobson; Bethesda, USA) and the immunosuppressant cisclosporin (an inhibitor of T cell proliferation by interfering with NFAT; Fabiola Martin; London, UK).

In the absence of efficient treatment for HAM/TSP, all these approaches merit further evaluation in clinical trials.

#### On the way towards an improved ATL therapy: from CHOP chemotherapy to AZT+IFN

Olivier Hermine (Paris, France) summarized a survey of ATL chemotherapy and showed that the current optimal regimen is AZT+IFNα [17]. In fact, it is essential not to provide general chemotherapy (CHOP) to first line presenting ATL patients because this treatment selects for a tumor clone with mutated p53. Overall response rate to AZT+IFNα was 66% including complete remissions. With 82% survival at 10 years after treatment, this therapy was particularly beneficial for acute ATL. Further improvements could include bortezomib (a proteasome inhibitor), anti-CD52 antibody (Campath), proapoptotic agents (Britta Moens; Leuven, Belgium) and consolidation with arsenic and IFNα. Ali Bazarbachi (Beirut, Lebanon) mentioned that AZT+IFNα has to be continuously provided to ATL patients to avoid relapse. Anti-viral therapy is also poorly efficient in the lymphoma subtype. Using the lck-Tax transgenic mouse model, he proposed a combination of arsenic trioxide (As_2_O_3_) and IFNα which contributes to the degradation of Tax. Triple therapy arsenic trioxide+AZT+IFNα merits further consideration to achieve complete response thereby allowing interruption of the AZT+IFNα treatment.

Concomitant with improved chemicals, it is also essential to identify biomarkers predictive of treatment outcome (Luiz Alcântara; Salvador, Brazil). Masao Seto (Nagoya, Japan) presented different genomic profiles in acute ATL having 3p amplifications and lymphoma type showing gains of chromosome 7 and 13q loss. Other prognostic markers included high IL5, CCR4 expression, p53 mutation, p16 deletion and sIL2α (Adrienne Philips; New York, USA).

## Concluding remarks

**Figure 2:**
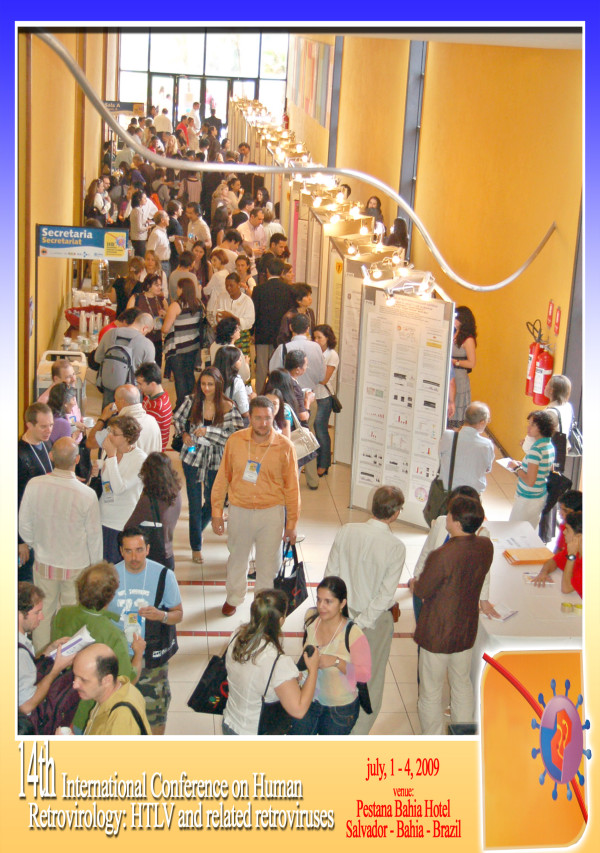
**The poster session fostered collaborations and stimulated new partnerships**.

After four days of meeting, the 14^th ^HTLV-1 conference concluded successfully with a robust exchange of new data and information (Fig. 2). As with all good conferences, the delegates departed perhaps with more new thoughtful questions to explore than with conclusive answers achieved. The 15^th ^HTLV-1 conference is scheduled to be in Leuven, Belgium in 2011 (to be co-organized by Annemieke Vandamme and Luc Willems). As with a meeting report from the 13^th ^conference [18], and this conference, we look forward to reporting the findings from the next conference. Goodbye Brazilian caipirinha, hello Belgian beer...

## Competing interests

The author declares that he has no competing interests.

## Authors' contributions

I collected the information and wrote the paper.
